# Laboratory based quantitative scoring system for pre-analytical quality indicators in arterial blood gas analysis

**DOI:** 10.1515/almed-2025-0179

**Published:** 2025-12-05

**Authors:** Tushar Sethi, Mohit Mehndiratta, Shiba Ansari, Ankita Gupta, Shashank Tripathi

**Affiliations:** Department of Biochemistry, University College of Medical Sciences & GTBH, Delhi, India; Department of Respiratory Medicine, University College of Medical Sciences & GTBH, Delhi, India; Department of Biostatistics, University College of Medical Sciences & GTBH, Delhi, India

**Keywords:** arterial blood gas (ABG) analysis, clinical biochemistry, emergency laboratory, laboratory quality assurance, pre-analytical errors, quality indicators (QIs)

## Abstract

**Objectives:**

Arterial blood gas (ABG) analysis is vital in emergency care setting for assessing respiratory and metabolic status, but its reliability is highly jeopardized by pre-analytical errors, which constitute up to 70 % of laboratory errors. This study evaluated pre-analytical quality indicators (QIs) of ABG testing by reviewing requisition forms and ABG samples at the emergency laboratory of a tertiary care hospital in Delhi, India.

**Methods:**

In this descriptive, questionnaire-based study, 205 ABG requisition forms and their corresponding samples collected over one year were assessed manually using a predefined checklist of pre-analytical quality indicators. Parameters included patient identification, requisition completeness, syringe type, tip sealing, labeling, sample volume, sample integrity, presence of clots, red cell sedimentation, and transport time (<30 min). Descriptive statistics were then applied.

**Results:**

The study results revealed major deficiencies. Documentation gaps included omission of collection time (97.07 % of all samples), ventilator/oxygen status mentioning (98.04 %), gender (9.27 %), age (6.34 %), and hospital unit (32.68 %). All samples were collected in inappropriate syringes; 89.27 % had inadequately sealed tips and 95.61 % lacked labeling. Transport delays (>30 min) occurred in 23.41 %, with clotting (14.15 %) and sedimentation (17.07 %) also noted. Inadequate volume (<0.3 mL) was found in 20 % of the total samples. Scores clustered at 8.5–10.5 (81 %), with mean=9.44 (SD=1.04); low (<8.0, 5 %) and outlier (>11.0, 3 %) scores were uncommon.

**Conclusions:**

Significant pre-analytical errors undermine ABG reliability, especially in documentation, collection, and transport. Standardized requisition forms, staff training, adherence to transport timelines, quality monitoring, and use of appropriate ABG syringes are essential to improve result accuracy and patient safety.

## Introduction

Arterial blood gas (ABG) analysis is an indispensable diagnostic tool in critical care and emergency medicine, offering vital insights into a patient’s respiratory function, acid–base balance and metabolic status. It plays a pivotal role in the diagnosis and management of conditions such as acute respiratory failure, metabolic disturbances, and various forms of shock, providing essential information for guiding timely and appropriate clinical interventions [1], 2]. ABG parameters typically include the partial pressure of oxygen (PaO_2_), partial pressure of carbon dioxide (PaCO_2_), blood pH, oxygen saturation (SaO_2_), and bicarbonate (HCO_3_^−^). These values accurately represent a patient’s oxygenation status (PaO_2_), ventilatory function (PaCO_2_), and acid–base equilibrium (pH and HCO_3_^−^) [3].

The reliability and clinical utility of ABG results are highly dependent on the quality of the pre-analytical phase, which encompasses all procedures performed prior to the actual analysis, including patient identification, sample collection, handling, transport, and storage [4], 5]. Studies consistently indicate that a significant proportion of laboratory errors – estimated to account for up to 70 % of total testing errors – occur in this phase, emphasizing the critical need for meticulous quality control protocols [6], 7]. Despite advances in laboratory automation, the pre-analytical phase remains heavily reliant on manual procedures, which increases the potential for human error [8].

Improper practices during this phase can lead to erroneous results and potentially harmful clinical decisions. For instance, inadequate anticoagulation, incorrect arterial puncture technique, sample contamination with air, delayed transport, or inappropriate storage can all compromise sample integrity [9]. Delays in analysis, for example, can lead to ongoing cellular metabolism, consuming oxygen and generating carbon dioxide, thereby decreasing pH and PaO_2_ and increasing PaCO_2_ [10]. Similarly, air contamination can falsely elevate PaO_2_ and reduce PaCO_2_, leading to misinterpretation of the patient’s respiratory status [11]. The use of excessive liquid heparin can result in dilutional effects, particularly on pH and electrolyte concentrations, such as ionized calcium and potassium [12]. Specific challenges in ABG testing also include incorrect sample volume, and inadequate documentation of clinical variables like ventilator use or supplemental oxygen [13].

To minimize errors, several quality indicators (QIs) have been established for the pre-analytical phase at the laboratory level. These include proper patient identification, appropriate heparin usage, and analysis within a recommended time frame – usually within 30 min of collection [14]. The implementation of these QIs is crucial for maintaining sample integrity and ensuring accurate results, particularly in high-stakes environments such as intensive care units and emergency departments. Previous studies have emphasized the importance of standardized protocols, training, and quality control measures to mitigate these errors [15], 16]. In this context, assessing both requisition form completeness and physical sample quality is critical for identifying gaps and improving ABG testing practices.

Therefore, while ABG analysis offers critical information for clinical decision-making in acutely ill patients, the accuracy of its results is highly susceptible to pre-analytical errors. This study was designed to evaluate key quality indicators in the pre-analytical phase of ABG analysis by examining requisition forms and corresponding blood gas samples. This comprehensive approach allowed for a detailed assessment of factors contributing to pre-analytical variability and identified targeted strategies for quality improvement, ultimately enhancing test reliability and contributing to better patient outcomes.

## Materials and methods

### Validation of instructional module as mentioned in Table 1

The validation process comprised a panel of evaluators from departments of respiratory medicine, anesthesia, biochemistry, laboratory medicine. The rating scales were sent to five evaluators. The process was undertaken to formulate a valid and user-friendly module which could adequately address the quality indicators of pre-analytical phase for ABG analysis. The evaluators received specific instructions to assess the important aspects, relevance etc. in the checklist attached. Scoring was conducted on a three-point scale ranging from 1 (strongly disagree) to 3 (strongly agree). Content validity ratios, content validity index (CVI) and modified kappa statistics were computed for validation.

**Table 1:** Content validation of ABG pre-analytical checklist.

Question	CVR	I-CVI	Pc	Kc	Interpretation
The checklist adequately covers the important aspects of the pre-analytical phase of ABG analysis	0.33	0.8	0.15	0.76	Excellent
The items included in the checklist are relevant to assessing the quality in ABG sample handling	1	1	0.03	1	Excellent

Scale-level indices: S-CVI/UA (universal agreement among experts) = 0.5; S-CVI/Avg (average content validity index at the scale level) = 0.9. CVR, content validity ratio; I-CVI, item content validity index; Pc, probability of chance agreement; Kc, modified kappa.

This descriptive, questionnaire-based study was conducted in the Emergency Laboratory of a tertiary care hospital affiliated with the Department of Biochemistry of our institution. Each ABG sample and its corresponding requisition form were assessed manually using a predefined checklist (Table 2), and an individual score was assigned for each item. Additionally, the percentage of errors was calculated for each QI across the total sample set.

**Table 2:** ABG sample checklist for laboratory (scoring system).

S. No.	Lab checklist item	Completed/Mentioned (score)	Not completed/not mentioned (score)
1	Patient’s name mentioned on the requisition slip	Yes (1)	No (0)
2	Patient’s gender mentioned on the requisition slip	Yes (0.5)	No (0)
3	Patient’s age mentioned on the requisition slip	Yes (0.5)	No (0)
4	Hospital registration no. mentioned on the requisition slip	Yes (1)	No (0)
5	ICU/ward/emergency mentioned on the requisition slip	Yes (0.5)	No (0)
6	Date of sample collection mentioned on the requisition slip	Yes (1)	No (0)
7	Time of sample collection mentioned on the requisition slip	Yes (1)	No (0)
8	Requisitioning doctor’s sign on the requisition slip	Yes (0.5)	No (0)
9	Requisitioning doctor’s stamp on the requisition slip	Yes (0.5)	No (0)
10	Mentioning whether patient is on ventilator/supplementary oxygen therapy	Yes (1)	No (0)
11	Sample adequate (≥0.3 mL)	Yes (1)	No (0)
12	Sample in correct ABG syringe	Yes (1)	No (0)
13	Tip of ABG syringe sealed properly/intact	Yes (1)	No (0)
14	Time taken (ideal=30 min) from collection to lab receipt	Yes (1)	No (0)
15	Visibly clotted sample	No (1)	Yes (0)
16	RBC sedimentation visible in sample/syringe	No (1)	Yes (0)
17	Syringe broken (sample spillage)	No (1)	Yes (0)
18	Labeling on syringe	Yes (0.5)	No (0)

### Study population and sample collection

A total of 205 ABG requisition forms and their corresponding blood samples were included in the study. These were selected using a simple random sampling method from all ABG samples received in the Emergency Laboratory during the study period of one year. Only samples collected for routine clinical care were included; research-specific samples or those with incomplete primary information were excluded.

### Data collection instrument and quality indicators

A predefined checklist was developed to independently assess each ABG sample and its accompanying requisition form. This checklist focused on key pre-analytical QIs, adapted from established laboratory guidelines and literature on ABG quality assurance. The QIs evaluated included:

– **Proper patient identification:** Verification of consistent patient identifiers (e.g., name, unique hospital ID number) on both the requisition form and the sample tube.
– **Use of appropriate anticoagulant (heparin):** Assessment of the sample tube to confirm the presence of heparin and the absence of clots, indicating proper anticoagulation.
– **Timely analysis:** Documentation of the time of sample collection (from the requisition form) and the time of analysis (from laboratory records) to ensure analysis occurred within the recommended 30 min of collection.
– **Completeness and accuracy of requisition form entries:** Evaluation of essential information on the form, including patient demographics, clinical context (e.g., oxygen therapy, ventilator settings), physician’s signature, and date and time of collection. Specific criteria for completeness and accuracy were defined prior to data collection.

### Scoring and data analysis

Each form and sample pair was meticulously evaluated against the predefined QIs. A scoring system ranging from 0 to 15 was developed, where higher scores indicated greater adherence to established quality standards for the pre-analytical phase as mentioned in Table 2. All collected data were meticulously compiled and organized using Microsoft Excel. Descriptive statistics were primarily used to interpret the compliance levels for each individual QI.

### Ethical statement

The research related to human use has been complied with all the relevant national regulations, institutional policies and in accordance the tenets of the Helsinki Declaration, and has been approved by the authors’ Institutional Review Board. Ethical approval for this study was obtained from the Institutional Ethics Committee prior to study initiation (Approval Number: IECHR-2024-67-11) and also this study was approved by research proposal advisory committee of the institute. The study design involved no direct interaction with patients, and all data collected were derived from anonymized laboratory records and samples. Stringent measures were taken to ensure patient confidentiality and data integrity by de-identifying all sensitive information prior to analysis. The study strictly adhered to the ethical guidelines for human research.

## Results

A total of 205 ABG requisition forms and their corresponding blood samples were analyzed for compliance with pre-analytical quality indicators. The evaluation revealed as mentioned in Table 3 significant deficiencies in documentation, sample collection, and handling practices. Notably, 97.07 % of requisition forms lacked information on the time of sample collection, and an equal proportion did not document ventilator or supplementary oxygen therapy status. Patient demographic details were inconsistently recorded, with gender missing in 9.27 % of forms, age in 6.34 %, and hospital unit in 32.68 % of forms, highlighting gaps in essential clinical information.

**Table 3:** Error rates for ABG pre-analytical quality indicators (n=205).

S. No.	Lab checklist item	Number compliant	Number compliant (errors)	Percentage error, %
1	Patient’s name mentioned on the requisition slip	200	5	2.44
2	Patient’s gender mentioned on the requisition slip	184	19	9.27
3	Patient’s age mentioned on the requisition slip	192	13	6.34
4	Hospital registration no. mentioned on the requisition slip	197	8	3.9
5	ICU/ward/emergency mentioned on the requisition slip	138	67	32.68
6	Date of sample collection mentioned on the requisition slip	203	2	0.98
7	Time of sample collection mentioned on the requisition slip	6	199	97.07
8	Requisitioning doctor’s sign on the requisition slip	198	7	3.41
9	Requisitioning doctor’s stamp on the requisition slip	201	4	1.95
10	Mentioning whether patient is on ventilator/supplementary oxygen therapy	4	201	98.04
11	Sample adequate (≥0.3 mL)	164	41	20.0
12	Sample in correct ABG syringe	0	205	100.0
13	Tip of ABG syringe sealed properly/intact	22	183	89.27
14	Time taken (ideal=30 min) from collection to lab receipt	157	48	23.41
15	Visibly clotted sample	176	29	14.15
16	RBC sedimentation visible in sample/syringe	170	35	17.07
17	Syringe broken (sample spillage)	189	16	7.8
18	Labeling on syringe	9	196	95.61

Assessment of sample collection and handling practices demonstrated considerable non-compliance with established standards. None of the 205 samples were collected in proper ABG syringes, and 89.27 % had inadequately sealed tips. Furthermore, 95.61 % of samples were improperly labeled, raising concerns about sample traceability and potential misidentification within the laboratory workflow. These deficiencies underscore the need for standardized collection protocols and staff training to ensure the integrity of ABG samples.

Transport and sample integrity were also areas of concern. Approximately 23.41 % of samples exceeded the recommended 30-min interval between collection and laboratory analysis, increasing the risk of ex vivo biochemical changes. Visible clotting was observed in 14.15 % of samples, while red blood cell sedimentation occurred in 17.07 %, indicating suboptimal anticoagulant mixing or delayed processing. Sample volume inadequacy (<0.3 mL) was noted in 20 % of cases, further compromising the accuracy of ABG measurements.

**Figure 1:**
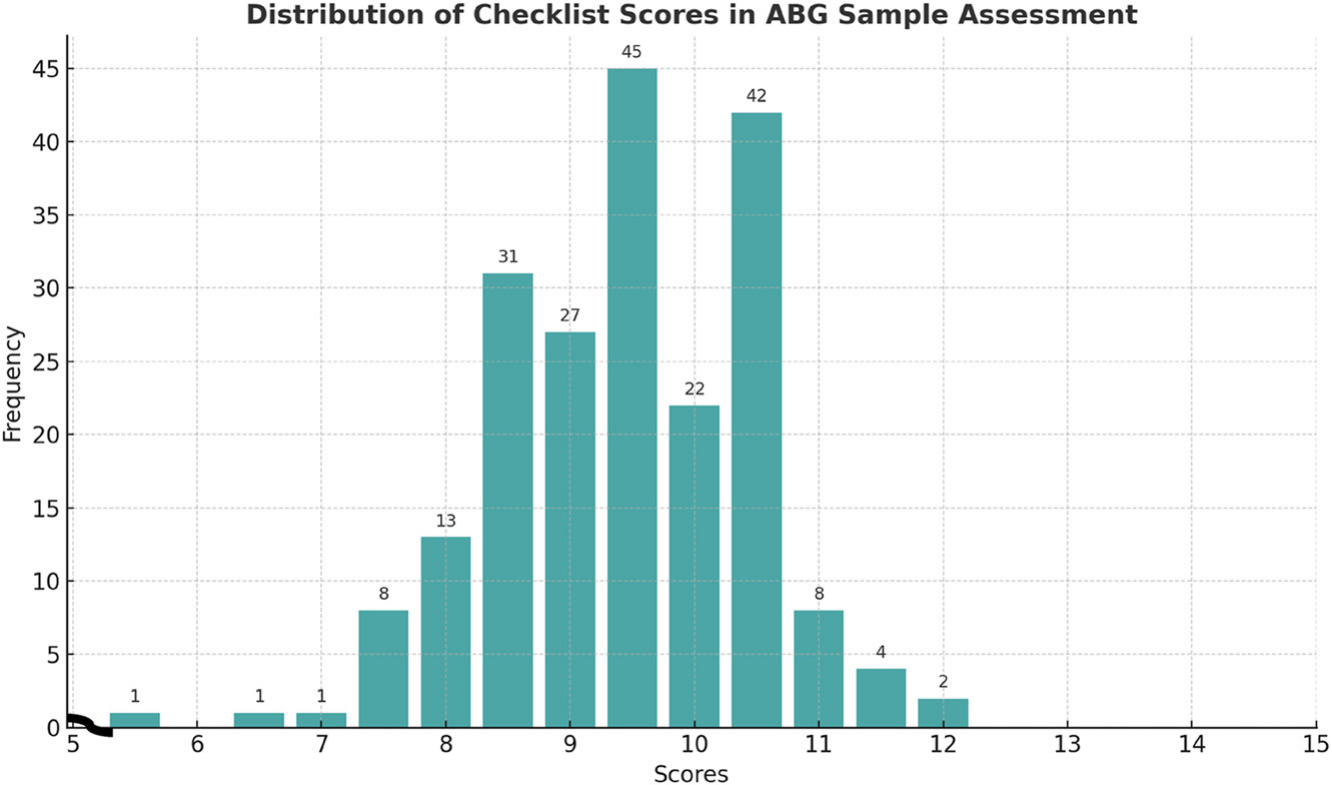
Distribution of individual sample scores.

The distribution of individual sample scores as shown in Figure 1 revealed that the majority clustered within the higher range (8.5–10.5), accounting for approximately 81 % of observations (167/205), indicating generally consistent performance. The mean was 9.44 (SD=1.04). Suboptimal scores (<8.0) were infrequent, representing ∼5 % of cases (11/205) and exceptionally high scores (>11.0, n=6, ∼3 %) were observed, suggesting occasional deviations in scoring or heightened stringency in evaluation. These findings highlight areas for targeted quality improvement and continuous monitoring to enhance pre-analytical performance in ABG testing.

## Discussion

The pre-analytical phase, is particularly critical in arterial blood gas (ABG) analysis, given the test’s inherent sensitivity to pre-analytical variables and its vital role in guiding immediate clinical decisions in critically ill patients [3].

Recent studies underscore the persistent vulnerability of arterial blood gas (ABG) measurements to pre-analytical errors, particularly when delays in analysis, temperature variations or air contamination are involved. For example, Çuhadar et al. found that among samples stored at 4 °C or 22 °C for 30–120 min, pO_2_ was the least stable parameter (especially at 4 °C), whereas at 22 °C pH and glucose remained stable up to 90 min and pCO_2_ up to 60 min – emphasizing the critical importance of both storage conditions and minimizing air bubbles [17]. More recently, a larger multi-patient study by Kristinsdottir et al. demonstrated that no ABG variable exceeded a pre-defined clinically relevant threshold at 30 min of delay, but pO_2_ did so by 45 min, thereby suggesting a practical “safe window” of ∼30 min for analysis under ideal conditions [18]. Together, these findings support the need for strict sample handling protocols – including immediate analysis when feasible, controlled temperature storage (preferably at room temperature if delay unavoidable), minimal air contamination, and standardized sample transport – to maintain result integrity and support accurate clinical decision-making.

Our study offers a novel, dual-focused assessment of both documentation quality and sample-handling integrity within the real-world setting of a high-volume emergency laboratory of a tertiary-care hospital. By analyzing 205 matched ABG requisition forms and corresponding samples manually, we provide actionable insights into how routine pre-analytical practices impact result reliability yielding specific evidence to inform targeted procedural improvements.

### Patient identification and documentation deficiencies

Accurate and complete patient identification and comprehensive clinical information on requisition forms are essential to ensure the validity of laboratory results and appropriate clinical interpretation [4]. A study done by Higgins C also revealed that adequate patient identification (e.g., name, ID, gender) is critical to avoid misinterpretation and miscommunication in ABG testing [19]. Our findings indicate that the majority of forms contained fundamental identifiers such as patient name, age, and hospital registration number, important parameters, including patient gender and hospital unit, were inconsistently documented. Moreover, incomplete documentation can disrupt seamless communication between laboratory and clinical teams regarding critical alert values, potentially delaying critical interventions and affecting patient outcomes.

### Suboptimal sample collection and handling practices

Proper sample collection, sealing, and labeling are imperative for preserving sample integrity and ensuring accurate test results [5]. Our analysis revealed notable deficiencies in these areas: a substantial 183 samples were inadequately sealed, significantly increasing the risk of air contamination and falsely elevated oxygen tensions [11]. Furthermore, 196 samples lacked proper labeling, raising serious concerns regarding sample misidentification and traceability within the laboratory workflow [5], 9]. Mostly when multiple ABG samples are received in the lab along with multiple requisition forms it is important for labeling to be present on syringes in order to correctly relate to its requisition form details. The absence of crucial clinical parameters, such as ventilator or supplemental oxygen status, on a subset of requisition forms further exacerbates the challenge, as this information is fundamental for accurate incorporation into the ABG analyzer and subsequent interpretation [8]. These findings underscore the urgent necessity for enhanced staff training and strict adherence to standardized sample collection protocols, aligning with recommendations from previous studies advocating for comprehensive training to mitigate pre-analytical errors [15], 16].

### Compromised sample integrity and delayed transport

Timely transport and prompt analysis of ABG samples are paramount to prevent *ex vivo* biochemical alterations. In this cohort, 48 samples exceeded the recommended maximum interval of 30 min from collection to laboratory receipt, putting them at significant risk of altered pH, PaO_2_, and PaCO_2_ values due to ongoing cellular metabolism [10]. Gupta et al. recommend that, unless critical evaluation of pO_2_ and pCO_2_ is required, analysis of the sample should be conducted within 30 min. Typically, samples arriving at the laboratory more than 30 min after collection were not accepted for analysis to prevent compromised results from degraded sample integrity [20]. Furthermore, 29 samples exhibited visible clotting, and 35 demonstrated red blood cell sedimentation, indicative of inadequate anticoagulant mixing or delayed processing. Such pre-analytical lapses can significantly impair result accuracy and potentially damage sensitive laboratory instrumentation [9], 10]. The observed rates of delayed transport and sample degradation highlight a critical area for immediate improvement in the pre-analytical workflow, given their direct impact on the reliability of results.

### Inadequate syringe use and sample volume

The adequacy of sample volume and the use of appropriate collection devices critically influence ABG accuracy. Our study found that 41 samples were collected with volumes below the recommended threshold (0.3 mL) [21]. Furthermore, none of the samples utilized dedicated ABG syringes, which are specifically designed to minimize air exposure and clot formation. Insufficient sample volume and the use of improper equipment have been consistently shown to adversely affect gas tension measurements, particularly PaCO_2_ and pH, thereby compromising the precision of clinical decision-making [12], 19]. Another study done by Baird G. stated insufficient volume of sample as a common pre-analytical error [22]. These findings suggest a need for re-evaluation of current practices and potentially the adoption of standardized ABG collection kits to ensure optimal sample quality.

### Limitations

This study provides valuable insights into the pre-analytical phase of ABG analysis at our institution, but it’s important to acknowledge its inherent limitations.

First, this was a single-center study conducted exclusively at the Emergency Laboratory of a Tertiary Care Hospital. The findings, while specific to our setting in Delhi, India, may not be broadly generalizable to other institutions. Different hospitals may have varying protocols, staffing levels, patient demographics, and laboratory equipment, all of which can influence the prevalence and types of pre-analytical errors.

Second, while we meticulously analyzed both requisition forms and corresponding physical samples, our study did not assess detailed clinical data on patients’ conditions or outcomes. This means we couldn’t directly correlate specific pre-analytical errors with their immediate clinical impact, such as delays in diagnosis, changes in patient management, or adverse events. Future research incorporating clinical outcomes would provide a more comprehensive understanding of the patient safety implications of pre-analytical errors.

Third, the study relied on a snapshot analysis of 205 samples over a limited time frame. While this sample size provided a robust assessment for our context, a larger, multicenter study conducted over an extended period would significantly strengthen the findings. Such a broader study could allow for trend analysis, identify seasonal variations in error rates, and enhance the statistical power to detect more subtle associations.

Finally, potential observer bias cannot be entirely excluded. The evaluation of sample handling and form completeness involved manual review by researchers. Although efforts were made to standardize the assessment criteria through a predefined checklist, individual interpretation could introduce some variability. Future studies could consider incorporating blinding mechanisms or automated data collection, where feasible, to mitigate this type of bias.

## Conclusions

The pre-analytical phase is undeniably pivotal in ensuring the accuracy and reliability of arterial blood gas (ABG) analysis, a critical diagnostic tool in acute care settings. Our study meticulously identified several key areas for improvement within this crucial phase. These include pervasive issues such as incomplete patient documentation on requisition forms, improper sample collection and handling techniques, delayed sample transport to the laboratory, and the inadequate use of appropriate syringes and adherence to recommended sample volumes.

Based on these compelling findings, we strongly advocate for the following interventions to comprehensively enhance pre-analytical quality in ABG testing. First, standardization of requisition forms is essential to mandate comprehensive patient demographics and critical clinical parameters, including ventilator settings and oxygen supplementation status [1], 15]. Second, rigorous, targeted training programs should be implemented for all staff involved in ABG sample collection and handling, emphasizing correct arterial puncture techniques, proper sample sealing, accurate labeling, and the critical importance of timely transport [5], 9].

Third, strict adherence to sample transport and processing timelines must be enforced, ensuring that all ABG samples are analyzed within 30 min of collection [10], 12]. Fourth, robust, continuous quality assurance programs should be maintained to proactively monitor pre-analytical variables and institute immediate corrective actions where necessary [1], 15], 16].

Finally, the mandatory use of appropriate, dedicated ABG syringes with strict adherence to recommended sample volume requirements is critical to significantly reduce analytical variability and improve result accuracy [12], 19]. Scores can also be checked periodically to track shifts toward higher ranges, particularly following staff training programs, to further reduce pre-analytical error levels.

Continued surveillance and corrective actions are recommended to maintain and elevate ABG quality indicator score. The scores can also be checked periodically for their shift towards higher ranges along with or after the staff training programmes are held to reduce the pre-analytical error levels. By prioritizing and systematically improving these pre-analytical processes, laboratories can significantly reduce the incidence of errors, thereby enhancing the overall quality and reliability of ABG results. Ultimately, these improvements will not only bolster the integrity of laboratory data but also directly support better-informed clinical decision-making, leading to optimized patient management and ultimately, improved patient outcomes in critical care environments.
